# Integrated Transcriptome Analysis of Human Visceral Adipocytes Unravels Dysregulated microRNA-Long Non-coding RNA-mRNA Networks in Obesity and Colorectal Cancer

**DOI:** 10.3389/fonc.2020.01089

**Published:** 2020-07-02

**Authors:** Sabrina Tait, Antonella Baldassarre, Andrea Masotti, Enrica Calura, Paolo Martini, Rosaria Varì, Beatrice Scazzocchio, Sandra Gessani, Manuela Del Cornò

**Affiliations:** ^1^Center for Gender-Specific Medicine, Istituto Superiore di Sanità, Rome, Italy; ^2^Bambino Gesù Children's Hospital-IRCCS, Research Laboratories, Rome, Italy; ^3^Department of Biology, University of Padua, Padua, Italy

**Keywords:** obesity, colorectal cancer, adipocyte, RNASeq, microRNAs, long non-coding RNAs, networks

## Abstract

Obesity, and the obesity-associated inflammation, represents a major risk factor for the development of chronic diseases, including colorectal cancer (CRC). Dysfunctional visceral adipose tissue (AT) is now recognized as key player in obesity-associated morbidities, although the biological processes underpinning the increased CRC risk in obese subjects are still a matter of debate. Recent findings have pointed to specific alterations in the expression pattern of non-coding RNAs (ncRNAs), such as microRNAs (miRNAs), and long non-coding RNAs (lncRNAs), as mechanisms underlying dysfunctional adipocyte phenotype in obesity. Nevertheless, the regulatory networks and interrelated processes relevant for adipocyte functions, that may contribute to a tumor-promoting microenvironment, are poorly known yet. To this end, based on RNA sequencing data, we identified lncRNAs and miRNAs, which are aberrantly expressed in visceral adipocytes from obese and CRC subjects, as compared to healthy lean control, and validated a panel of modulated ncRNAs by real-time qPCR. Furthermore, by combining the differentially expressed lncRNA and miRNA profiles with the transcriptome analysis dataset of adipocytes from lean and obese subjects affected or not by CRC, lncRNA–miRNA–mRNA adipocyte networks were defined for obese and CRC subjects. This analysis highlighted several ncRNAs modulation that are common to both obesity and CRC or unique of each disorder. Functional enrichment analysis of network-related mRNA targets, revealed dysregulated pathways associated with metabolic processes, lipid and energy metabolism, inflammation, and cancer. Moreover, adipocytes from obese subjects affected by CRC exhibited a higher complexity, in terms of number of genes, lncRNAs, miRNAs, and biological processes found to be dysregulated, providing evidence that the transcriptional and post-transcriptional program of adipocytes from CRC patients is deeply affected by obesity. Overall, this study adds further evidence for a central role of visceral adipocyte dysfunctions in the obesity–cancer relationship.

## Introduction

The increase of obesity is a major health problem afflicting nowadays adults and children worldwide (1). Obesity is a complex condition, characterized by excessive expansion and functional alteration of white adipose tissue (AT), that increases the risk of life threatening diseases such as cardiovascular disease, diabetes and cancer, including colorectal cancer (CRC). Indeed, white AT, particularly visceral fat, is a complex endocrine and immunocompetent organ, homing adipocytes and resident immune cells, exhibiting secretory as well as immunological, metabolic, and endocrine regulatory activities and playing a central role in obesity-associated morbidities (2). Its functional units, the adipocytes, produce and secrete a large array of mediators including cytokines/chemokines, extracellular matrix proteins, hormones, growth and angiogenic factors that influence, either locally or systemically, a variety of physiological and pathological processes, such as immune functions, cell proliferation, migration, angiogenesis (3, 4). In addition of being an established risk factor (5), excess adiposity is also associated with CRC worse outcomes (6, 7), although the mechanisms underlying the detrimental link between obesity and CRC are complex and not yet precisely defined. In this respect, it has been postulated that this association may be due to the large spectrum of cytokines and metabolites that are produced by AT showing pro-inflammatory and cancer prone features. Moreover, obesity-related metabolic alterations (i.e., triggering of insulin resistance, impairment in lipid metabolism, endocrinologic changes and oxidative stress) may contribute to CRC initiation and progression (8). More recently, emerging evidence point to the role of non-coding RNAs (ncRNAs) in many obesity-related disorders including cardiovascular and metabolic diseases, inflammation, and cancer (9), and more specifically in CRC (10).

NcRNAs are transcripts that are not translated into proteins. They are present in all organisms, where they regulate gene expression and, therefore, biological processes, at the transcriptional and post-transcriptional level (11). Multiple types of regulatory ncRNAs are emerging as key elements of cellular homeostasis and diseases. Among these long ncRNAs (lncRNAs) (>200 nts) and small ncRNAs (<200 nts), such as microRNAs (miRNAs), small interfering-, Piwi interacting-, small nucleolar-, small nuclear-, extracellular-RNAs, are arbitrarily classified according to their nucleotide length (12). Among them, microRNAs (miRNAs) are evolutionarily conserved small ncRNAs (18–25 nt in length) playing a crucial role in cell transcriptional regulation (13, 14). Their expression correlates with different obesity relevant parameters, such as body mass index (BMI), adipocyte size and metabolic parameters, highlighting important regulatory role in obesity (15–18). The importance of miRNAs in mediating the initiation, growth, and development of CRC was also reported (19). In contrast with small ncRNAs, lncRNAs undergo post-transcriptional modifications, such as polyadenylation and splicing, although they lack protein-coding capacity (20). They are emerging as miRNA sponges and inhibitors, thus releasing downstream genes from the miRNA control (21). Furthermore, lncRNAs can also interact with DNA, RNA and proteins, overall regulating gene expression and epigenetic status (12). Accumulating evidence has revealed that the expression of lncRNAs is involved in the occurrence and development of many major diseases, including human cancers (22, 23), and that lncRNA-miRNA-mRNA networks are specifically associated with CRC (24). High-throughput methods and bioinformatics approaches have significantly contributed to the identification of new transcripts, including ncRNAs. However, only few studies have described miRNAs and lncRNAs in human AT under obesity (9, 25–27). Moreover, no studies have reported the expression of miRNAs and lncRNAs in AT from CRC patients. In this regard, we recently reported that obesity and CRC, conditions characterized by the common denominator of inflammation, are associated with changes in the transcriptional program of adipocytes mostly involving pathways and biological processes linked to fibrosis, inflammation and metabolism of pyruvate, lipids, and glucose (28). In this study, we analyzed the ncRNA expression profiles, specifically miRNAs and lncRNAs, of lean and obese subjects affected or not by CRC, by RNASeq/Small RNASeq analysis. This approach allowed to highlight changes in adipocyte miRNA and lncRNA profiles that are specifically associated with obesity or CRC, or shared by both conditions. Finally, by integrating bioinformatics prediction, functional enrichment analysis, and data on differential mRNA expression previously described (28), we identified lncRNA-miRNA-mRNA regulatory networks and defined multiple pathways characterizing visceral adipocytes, that are altered in obesity and/or CRC. Overall, this might contribute to set the basis for a more tumor-prone microenvironment, thus adding further evidence for the central role of AT functional alterations in linking obesity to cancer.

## Methods

### Ethics Statement

Investigation has been conducted in accordance with the ethical standards and with the Declaration of Helsinki, and according to national and international guidelines. It was approved by the institutional review board of Istituto Superiore di Sanità. All enrolled subjects were provided with complete information about the study and asked to sign an informed consent.

### Patient and Sample Collection

As previously described (28), “human visceral adipose tissue (VAT) was collected from age-matched lean and obese subjects undergoing abdominal surgery or laparoscopy for benign (i.e., gallbladder disease without icterus, umbilical hernia, and uterine fibromatosis) or CRC conditions (histologically proved primary colon adenocarcinoma, stage TNM 0–III). The exclusion's criteria were: clinical evidence of active infection, recent (within 14 days) use of antibiotics/anti-inflammatory drugs, pregnancy, hormonal therapies, severe mental illness, autoimmune diseases, family history of cancer, others neoplastic diseases. In the normal weight group, the BMI range was 20–25 Kg/m^2^. In the obese group the BMI was ≥ 30 Kg/m^2^, and waist circumference > 95 cm for men and > 80 cm for women. The total number of subjects was six/category.”

### Adipocyte Isolation, RNA Preparation and Sequencing

Adipocytes were isolated from human VAT as previously described (29). Total RNA was isolated with Total RNA Purification Plus Kit (Norgen Biotek, Canada). RNA quality and quantity was assessed by Agilent 2,100 Bioanalyzer and samples stored at −80°C until use. Total RNA (2 μg) was used to prepare the library for Illumina sequencing (Illumina TruSeq Small RNA Sample Preparation). Single-end reads (>10 M reads per sample) were produced by Illumina HiSeq 2000.

### RNASeq Data Preprocessing and Differential Expression Analysis

Libraries were then processed with Illumina cBot for cluster generation on the flowcell, following the manufacturer's instructions and sequenced on single-end mode at the multiplexing level requested on HiSeq2000 (Illumina, San Diego, CA). The CASAVA 1.8.2 version of the Illumina pipeline was used to process raw data for both format conversion and de-multiplexing. Adapters were removed and low-quality bases were trimmed by the script TrimGalore. Per sample, per read and per base quality of raw sequence data have been assessed with FastQC version 0.11.3 (http://www.bioinformatics.babraham.ac.uk/projects/fastqc/) and all the included samples passed the initial quality checks. All the sequencing data had all the range of the per base quality values into very good quality calls, lower than the 0.02% of the total sequences showed a per sequence low quality score and no adapter content. Thus, no quality trimming where performed during preprocessing. The percentage of mapped reads resulted high with the mean value of 97.5% (min 94.08% and max 98.41%).

The transcriptome reconstruction was performed as previously described (28). Re-annotation of previously unknown transcripts was performed using the bioMart package (30) into R 3.6 (31), querying available Ensemble transcript IDs and retrieving Gene Names, Entrez gene IDs, gene and transcript biotypes thus allowing the identification of a higher number of lncRNAs. Multiple testing controlling procedure was applied following Benjamini & Hochberg method hereafter referred as False Discovery Rate (FDR). We then extracted the list of differentially expressed lncRNAs (DEL) with a False Discovery Rate (FDR) ≤ 0.05. For small RNASeq analysis, raw reads where pre-processed using cutadatapt 1.9.1 (http://code.google.com/p/cutadapt/) and reads shorter than 17 bases were excluded. MiRNA expression quantification was carried out using MirDeep2 (version 2.0.0.8, Bowtie version 1.1.2) (32) using hg38.p2 genome version and 79 Ensembl version. MiRNA mature/hairpin sequences were downloaded from Mirbase 21 version (33), then, raw counts were filtered to keep only miRNA with more or equal to 10 reads in at least one sample. MiRNA expression was normalized with upper quantile normalization (EDASeq version 2.10.0) (34) while differential expression (all comparisons) was computed using edgeR (3.18.1) (35) from raw counts. All analyses were carried out in R and Bioconductor 3.5 version (https://bioconductor.org). Due to the limited differential expression of miRNAs (DEM), the threshold of FDR was set ≤ 0.06. For small RNA sequencing, six biological replicates per category were prepared and the raw sequence data are available from the NCBI Sequence Read Archive (SRA) (http://www.ncbi.nlm.nih.gov/sra) under accession number SRA: PRJNA632999. For long RNA sequencing, we employed the RNASeq datasets previously published and available under accession number SRA: PRJNA508473 (28).

### mRNA-miRNA-lncRNA Regulation Network Construction

Target genes of the identified differentially expressed miRNAs (DEM) were searched in the TarBase v.8 (36) and miRTarBase 7.0 (37) databases which feature up-to-date experimentally validated miRNA-targets interactions. Interactions between DEL and DEM were retrieved in both the DIANA-LncBase v2.0 database (38), using the prediction module and a score ≥ 0.6 as cut-off, and the ENCORI database (39) featuring experimentally verified RNA-RNA interactions. The ENCORI database was also used to search for DEL-mRNA verified interactions. The overall targets of DEM and DEL were filtered against the lists of differentially expressed transcripts (DET) and integrated to define specific mRNA-miRNA-lncRNA interactions networks for each condition. The Cytoscape software (40) was used to visualize the obtained networks.

### Functional Analysis

The cumulative list of DEM and DEL targets within the DET of each condition was explored for significantly enriched pathways with the Cytoscape plug-in ClueGO and CluePEDIA (41) querying the KEGG, WikiPathways and Reactome databases. Default settings were used for the pathways selection, connectivity and grouping. A two-sided enrichement analysis was performed, adjusting the *p*-values with the Benjamini-Hochberg correction and considering significant only pathways with *p* < 0.05.

### Real-Time qPCR Validation of Differentially Expressed lncRNAs and miRNAs

Twelve candidate ncRNAs, found differentially expressed by RNASeq, were selected for validation by real time qPCR (RT-qPCR). The validation of lncRNA expression was performed by qPCR using SYBRGreen assays (Supplemental Table 1). The synthesis of cDNA was performed by using 300–500 ng of total RNA in 20 μL reaction volume using the Superscript III kit (Thermofisher Scientific) following the manufacturer's instructions. The reverse transcription conditions were as follows: 5 min at 25°C, 60 min at 50°C, and 15 min at 70°C. cDNA was mixed with 2 × SensiFast SYBR low rox (Bioline), lncRNA expression values were normalized to the expression of GUSB as the endogenous control. For the validation of miRNA expression levels, we started the reverse transcription of 6 miRNAs by using 2 μl (5 ng/μl) of total RNA with the miRCURY LNA RT Kit (Qiagen). The reverse transcription conditions were as follows: 60 min at 42°C and 5 min at 95°C. cDNA was mixed with 2 × miRCURY SYBR Green Master Mix (Qiagen) following the manufacturer's instructions. The expression values of miRNAs were normalized to the expression of let-7a-5p as the endogenous control. For each sample, the relative expression level was determined according to the 2 –ΔΔCT method after running the samples on a QuantStudio 12 K Flex Real-Time PCR System (Thermofisher Scientific) following the manufacturer's instructions. For each sample, the relative gene expression level was determined according to the 2 –ΔΔCT method. Statistical comparisons of means from six biological replicates, matched with RNASeq analysis, was performed between the various subject groups (five for the NwCRC group) by one-way analysis of variance (ANOVA) with LSD *post hoc* tests by using SPSS software (Ver.20). Differences were considered statistically significant when *p*-values were ≤ 0.05. Analysis of correlation between qPCR and RNASeq data was performed by Spearman's rank test setting significance at *p* < 0.05.

## Results

### Long and Small RNA Sequencing Analysis Identify Differentially Expressed lncRNAs and miRNAs That Are Associated With Obesity and/or CRC

**Figure 1:**
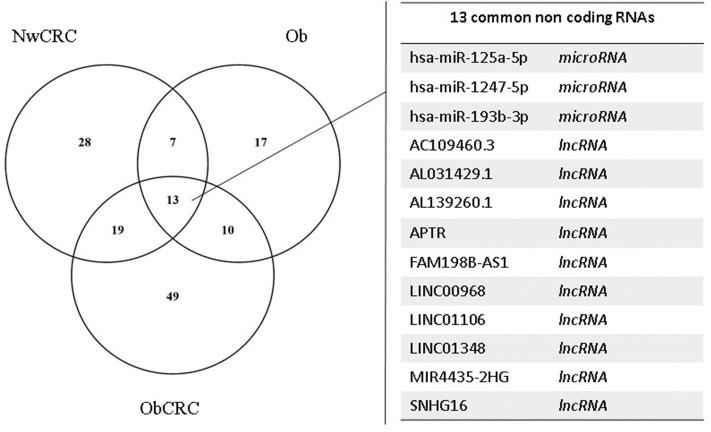
Analysis of lncRNAs and miRNAs shared by obese and CRC-affected individuals or unique for each condition. Venn diagram showing unique or shared ncRNAs resulting by the comparison of DEL and DEM from all pathological conditions vs. healthy lean subjects. Each comparison is represented by a circle. The numbers in the region of the overlapping circles indicate the ncRNAs that are expressed in two or more conditions. The complete list of the 13 ncRNAs shared by obesity and CRC is shown on the right.

We have previously analyzed the transcriptome profiles of human adipocytes isolated from visceral AT (VAT) biopsies obtained from healthy control lean (normal weight, Nw) and obese (Ob) subjects, or CRC patients (normal weight or obese, NwCRC, and ObCRC, respectively), by RNA sequencing (28). Along with the protein coding transcripts, the long RNASeq analysis detected also a total of 90 differentially expressed lncRNAs (DEL, FDR ≤ 0.05), 35 of which were novel transcripts (Table 1). In NwCRC subjects, 45 DEL were found dysregulated (11 downregulated, 33 upregulated and one DEL with two transcripts inversely modulated, NUTM2A-AS1) compared to Nw healthy controls. In Ob group, we found 27 DEL (3 downregulated, 23 upregulated and one DEL with two inversely modulated transcripts, RASSF8-AS1). Finally, when comparing ObCRC group with the control lean group, a total of 52 DEL, including 13 downregulated, 38 upregulated and one with three transcripts (MIR4435-2HG, one up- and two downregulated), were found. Among the overall 90 DEL, 10 were shared by all the three subject categories (AC109460.3, AL031429.1, AL139260.1, APTR, FAM198B-AS1, LINC00968, LINC01106, LINC01348, MIR4435-2HG, SNHG16), 6 were shared by NwCRC and Ob patients (AC008105.3, AC021092.1, HIF1A-AS1, LINC00926, RASSF8-AS1, ZNF883), 12 were shared by NwCRC and ObCRC patients (AC009022.1, AC010457.1, AC016582.2, AC068888.1, AL356056.1, AP000317.2, FAM27E3, MINCR, MIR100HG, SLC14A2-AS1, STAG3L5P-PVRIG2P-PILRB, TPTEP1), and only one was shared by Ob and ObCRC patients (AC022007.1). On the other hand, a number of lncRNAs were selectively modulated in each subject category, with the ObCRC group exhibiting the highest number of specific DEL (Table 1). In parallel, small RNASeq analysis revealed a total of 58 differentially expressed miRNAs (DEM, FDR ≤ 0.06) in adipocytes of NwCRC, Ob, and ObCRC subjects compared to Nw individuals (Table 2). Specifically, 22 DEM were found in NwCRC (12 upregulated and 10 downregulated), 20 DEM were detected in Ob subjects (13 upregulated and 7 downregulated), while the comparison of ObCRC with Nw control revealed a higher number of dysregulated miRNAs (39 DEM, 20 upregulated and 19 downregulated), suggesting that the conditions of obesity and CRC interact concurrently, thus influencing the miRNA expression profile in adipocyte from ObCRC subjects. Among the overall modulated 58 DEM, only 3 were common to all group of subjects (miR-1247-5p, miR-125a-5p, miR-193b-3p), 7 were shared by NwCRC and ObCRC subjects (miR-125b-1-3p, miR-22-5p, miR-29b-2-5p, miR-4455, miR-452-5p, miR-7706, miR-98-5p), 9 were shared by ObCRC and Ob subjects (let-7e-3p, miR-1287-5p, miR-152-3p, miR-181c-5p, miR-181d-5p, miR-185-5p, miR-24-3p, miR-34a-5p, miR-421), while only one was common to NwCRC and Ob subjects (miR-345-5p). As regards the subject group-specific DEM, again the ObCRC category exhibited the highest number of selectively dysregulated miRNAs (Table 2). A Venn diagram was then generated to discover the common or unique lncRNAs and miRNAs among the three experimental groups (Ob, NwCRC, and ObCRC subjects) (Figure 1). By intersecting DEL and DEM data from the three comparisons (NwCRC, Ob and ObCRC individuals compared to Nw subjects), 13 ncRNAs were found to be shared between cancer and obese conditions. The identification of these differentially expressed ncRNAs, likely involved directly in creating a tumor-promoting microenvironment, may provide clues on the epigenetic mechanisms by which obesity favor CRC onset, as well as on how CRC development in obesity differs from that in lean individuals.

**Table 1:** Differentially expressed lncRNAs in normal weight affected by CRC (NwCRC), obese (Ob), and obese affected by CRC (ObCRC) individuals vs. healthy lean control.

		**Log**_****2****_**FC (FDR** **≤** **5%)**		
**Gene Name**	**Entrez Gene ID**	**NwCRC**	**Ob**	**ObCRC**
Novel lncRNA				
AC004241.1		4.4723		
AC004477.3		1.9133		
AC006504.5		2.3449		
AC007098.1				6.6690
AC008105.3		4.2391	4.4356	
AC009022.1		8.7676		8.7671
AC010457.1		2.2489		2.8844
AC016582.2		5.1047		
AC021092.1		1.1310	1.0531	
AC022007.1			9.0501	9.3198
AC023421.1				5.4800
AC061992.1		−4.2812		
AC068473.5		1.4616		
AC068888.1		7.4252		8.3630
AC084757.3				−2.9022
AC092279.1		−5.9393		
AC099518.3				−7.8032
AC109460.3		6.9064	6.8707	6.2148
AC114956.3				2.6267
AC139256.1			2.8280	
AC144548.1				−1.0320
AC141930.1		3.2551		
AL031429.1		3.3503	3.0813	4.1653
AL078612.1				2.7046
AL138828.1				2.3053
AL138963.4				2.2073
AL139260.1		7.4131	6.2839	7.3094
AC016582.1				5.2659
AL161772.1				1.9376
AL355607.2				1.9507
AL356056.1		5.4758		6.9848
AL591848.3		1.5321		
AP000317.2				−11.2572
AP000790.1				6.1063
FP236383.3				8.4412
Known lncRNA				
AGAP11	119385			1.8547
APTR	100505854	3.3044	4.0329	2.8546
ARHGEF7-AS2	100874238			4.6621
BCYRN1	618			3.1417
CFLAR-AS1	65072	−6.3218		
DLGAP1-AS1	649446		1.4003	
FAM198B-AS1	285505	3.8088	4.4575	4.6582
FAM27E3	100131997			3.9401
FOXP4-AS1	101060264			6.3680
H19	283120	−22.6445		
HIF1A-AS1	100750246	3.7185	2.9894	
HOXB-AS3	404266		−7.1867	
LINC00486	285045			6.9600
LINC00926	283663	4.5816	4.6596	
LINC00968	100507632	8.9218	8.3768	8.4248
LINC01106	151009	19.5614	19.7102	22.0251
LINC01106	151009	20.9418	20.6120	19.8254
LINC01140	339524	−3.7389		
LINC01140	339524	−3.5970		
LINC01184	644873			−8.4369
LINC01239	441389			−2.1368
LINC01291	102724515			−21.0782
LINC01348	731656	8.9118	8.2512	7.3795
LINC01619	256021			−8.8258
LNCOG	105369848		1.8031	
LUCAT1	100505994		9.5244	
MALINC1	100505636		2.0735	
MAP4K3-DT	728730			5.1283
MINCR	100507316	3.7292		4.1920
MIR100HG	399959	−8.2382		−21.7228
MIR100HG	399959			−9.8115
MIR3142HG	107075116		3.1109	
MIR4435-2HG	541471	9.2841	9.7786	9.1337
MIR4435-2HG	541471			−9.4517
MIR4435-2HG	541471			−8.6134
MSC-AS1	100132891	−23.2039		
NUTM2A-AS1	728190	−6.5505		
NUTM2A-AS1	728190	9.2961		
OLMALINC	90271			−2.9871
PGM5P3-AS1	101929127	−7.6345		
RASSF8-AS1	100506451	19.9364	22.0934	
RASSF8-AS1	100506451		−9.9462	
SCAT8	112935969	−3.9716		
SLC14A2-AS1	101927980	6.4340		9.1498
SNHG16	100507246	4.6908	4.6101	5.3027
SNHG29	125144			−7.6114
SNORD3C	780853		6.8661	
STAG3L5P-PVRIG2P-PILRB	101752399			20.8685
TMEM161B-AS1	100505894			4.6379
TPRG1-AS1	100874043		−1.6872	
TPTEP1	387590			7.4005
TPT1-AS1	100190939	−6.8438		
UBA6-AS1	550112			−8.0421
USP9Y	8287	8.9477		
XIST	7503			−25.1297
ZFAS1	441951		−5.2589	
ZNF295-AS1	150142			1.8551
ZNF883	169834	6.9421	7.4847	

**Table 2:** Differentially expressed miRNAs in normal weight affected by CRC (NwCRC), obese (Ob), and obese affected by CRC (ObCRC) individuals vs. healthy lean control.

**miRNA**	**Log**_****2****_**FC (FDR** **≤** **6%)**		
	**NwCRC**	**Ob**	**ObCRC**
let-7c-5p		0.8158	
let-7e-3p		−0.5273	−0.5072
let-7f-5p	0.6367		
let-7i-3p			−0.7816
miR-100-5p			−0.8363
miR-107			0.4816
miR-10b-3p			0.9854
miR-10b-5p			1.0690
miR-1246	1.4342		
miR-1247-5p	−1.1469	−1.2365	−1.0182
miR-125a-5p	−0.6525	−0.6889	−0.7270
miR-125b-1-3p	−1.1204		−1.1041
miR-1287-5p		1.1111	1.2275
miR-1296-5p		−0.9913	
miR-1299			1.2495
miR-1323			−1.4956
miR-144-5p		1.3677	
miR-152-3p		0.6263	0.6534
miR-181c-3p		1.1703	
miR-181c-5p		1.2190	1.2047
miR-181d-5p		1.2244	1.2720
miR-185-5p		0.784	0.8778
miR-193b-3p	−0.7937	−0.7410	−0.7744
miR-22-5p	0.6981		0.7611
miR-24-3p		0.6745	0.7660
miR-28-5p			0.4959
miR-29b-2-5p	0.6554		0.7330
miR-29b-3p	0.9662		
miR-30c-5p			−0.6740
miR-3182	1.2731		
miR-328-3p	−0.8400		
miR-33b-3p	−1.2430		
miR-345-5p	−0.6699	−0.8146	
miR-34a-5p		0.9133	1.1756
miR-361-3p		−0.4835	
miR-3622a-5p	−1.249		
miR-374a-3p	0.7650		
miR-374b-5p	0.9486		
miR-378f			−1.0918
miR-421		0.8311	0.8296
miR-4455	−1.3104		−1.2491
miR-451a		1.2621	
miR-452-5p	0.6354		0.7434
miR-483-5p		1.0648	
miR-508-3p			1.1567
miR-512-3p			−1.3976
miR-515-5p			−1.1957
miR-516a-5p			−1.4569
miR-516b-5p			−1.3241
miR-517a-3p			−1.5517
miR-517b-3p			−1.5517
miR-548az-5p			1.3631
miR-598-3p	0.9845		
miR-664a-3p	0.9304		
miR-7706	−0.8991		−0.6495
miR-92a-3p			−0.5834
miR-98-5p	0.7211		0.6373
miR-99a-3p			0.5715

### Identification of Target Genes Regulated by Differentially Expressed miRNAs

**Figure 2:**
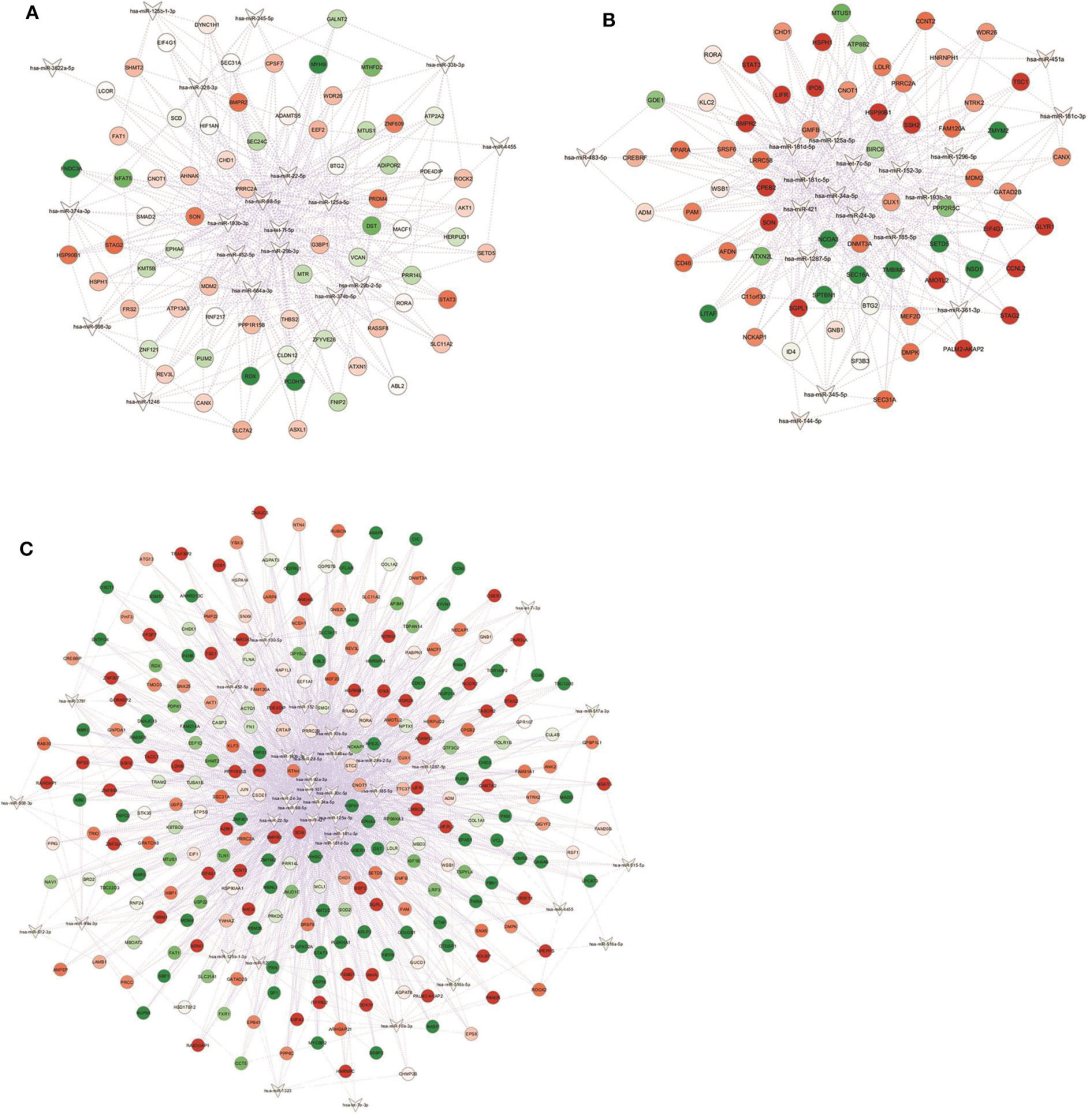
miRNA-gene targets regulatory networks. Interaction networks between deregulated miRNAs (DEM; V-shape) and validated mRNA targets among modulated genes (DET; circles) in **(A)** NwCRC, **(B)** Ob, and **(C)** ObCRC patients in comparison to healthy lean subjects. Every node represents one gene, and each edge represents the interaction between genes. Only nodes with a number of directed edges ≥ 5 are shown (see Supplemental Table 2 for the extended network). Shades of green and red indicate, respectively, down- or up-regulated DEM/DET.

To investigate the potential involvement of the aforementioned DEM in the pathogenic events related to obesity and/or CRC, we next analyzed dysregulated miRNAs and validated consistency of differential expression of their targets. For each identified DEM, we extracted the list of experimentally validated mRNA targets from TarBase and miRTarBase repositories. Based on our previously obtained gene expression dataset (28), we considered only those targets included in the list of differentially expressed transcripts (DET). We then assembled an interaction network between DEM and their target genes for each group (Figure 2). The complete list of DEM-DET interactions for each condition is reported in Supplemental Table 2.

In detail, interaction analysis showed 713 nodes (21 DEM and 692 target DET) and 1,669 edges in the NwCRC network (Supplemental Table 2), with two DEM having a number of directed edges ≥ 200 (hsa-let-7f-5p and hsa-miR-98-5p) and five DEM having < 200 ≥ 100 directed edges (hsa-miR-193b-3p, hsa-miR-29b-3p, hsa-miR-125a-5p, hsa-miR-22-5p, and hsa-miR-374b-5p). Among the modulated genes, BTG2, and SON genes were the target of 10 DEM and other 33 DET interacted with more than five DEM. In the interaction network of Ob subjects, 808 nodes (20 DEM and 788 DET) and 1,759 edges were found (Supplemental Table 2), with hsa-miR-34a-5p having 420 directed edges and six DEM having over 100 directed edges (hsa-let-7c-5p, hsa-miR-24-3p, hsa-miR-193b-3p, hsa-miR-185-5p, hsa-miR-181c-5p, hsa-miR-125a-5p). SON was the target genes of 10 DEM and 33 DET interacted with more than five DEM. In ObCRC subjects 1,056 nodes (37 DEM and 1,019 DET) and 3,449 edges were found (Supplemental Table 2). hsa-miR-34a-5p and hsa-miR-107 had, respectively, 464 and 357 targets, four DEM had over 200 direct edges (hsa-miR-92a-3p, hsa-miR-24-3p, hsa-miR-98-5p, hsa-miR-30c-5p), seven DEM had < 200 ≥ 100 directed edges (hsa-miR-10b-5p, hsa-miR-193b-3p, hsa-miR-22-5p, hsa-miR-125a-5p, hsa-miR-185-5p, hsa-miR-181c-5p, hsa-miR-181d-5p). The top interacting DET was again SON and other 19 DET had more than 10 directed edges.

### Identification of Target Genes and microRNAs Regulated by Differentially Expressed lncRNAs

In addition to the miRNA regulatory networks, the dysregulation of lncRNA expression was recently associated with obesity and CRC (27, 42). Therefore, we constructed lncRNA-mRNA regulatory networks through an integrated analysis of the new identified DEL and the previously described DET (28), for each category of subjects.

**Figure 3:**
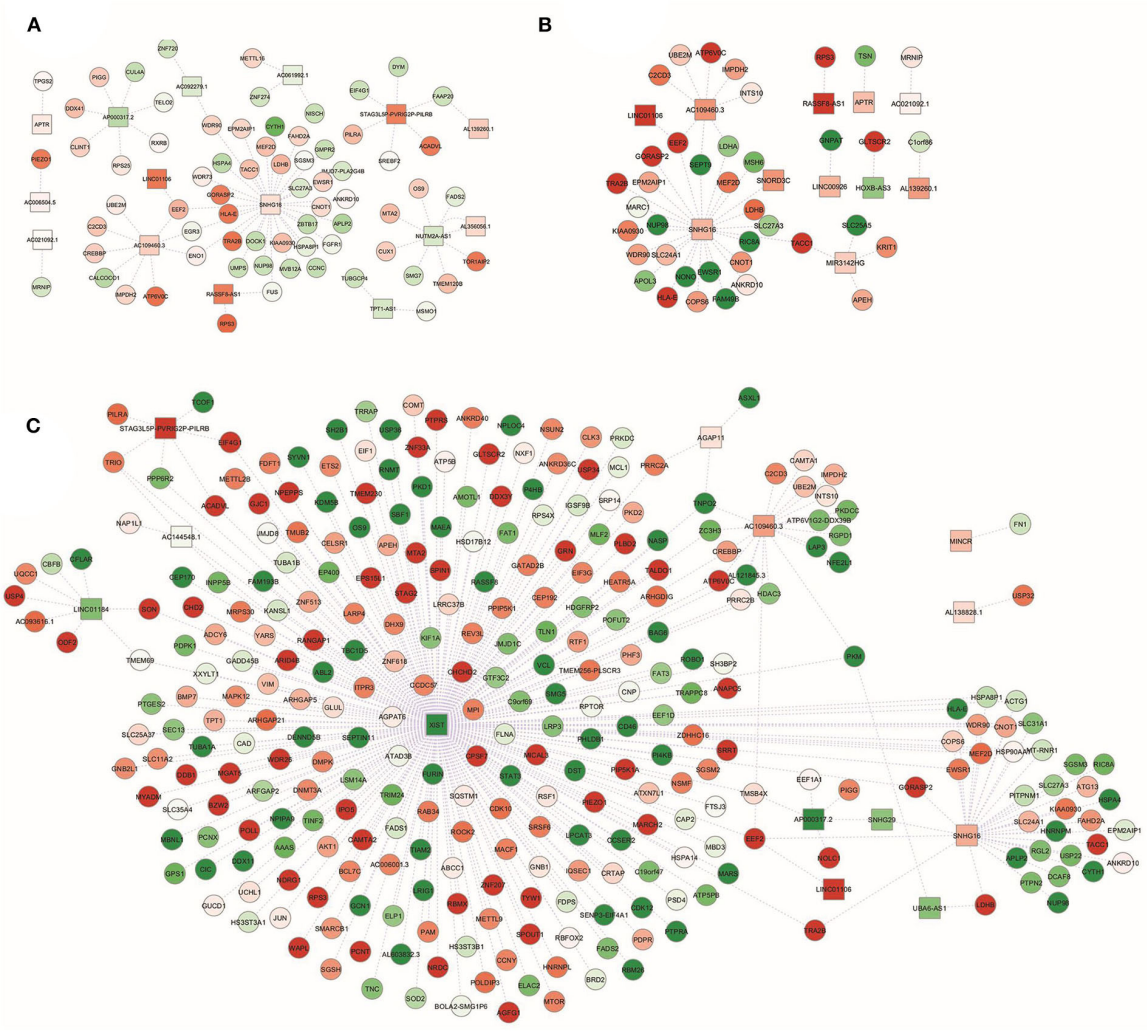
lncRNA-gene targets regulatory networks. Interaction networks between deregulated lncRNAs (DEL; squares) and validated mRNA targets among modulated genes (DET; circles) in **(A)** NwCRC, **(B)** Ob, and **(C)** ObCRC patients in comparison to healthy lean subjects. Every node represents one gene, and each edge represents the interaction between genes. Shades of green and red indicate, respectively, down- or up-regulated DEL/DET.

As shown in Figure 3, only for a subgroup of DEL at least one experimentally validated interaction was found in the ENCORI database. In particular, in NwCRC subjects, the up-regulated DEL SNHG16, AC109460.3, NUTM2A-AS1, and STAG3L5P-PVRIG2P-PILRB, as well as the down-regulated AP000317.2, were relevant hubs each interacting with more than three DET (Figure 3A). In Ob subjects, main nodes were represented by the up-regulated SNHG16, AC109460.3, and MIR3142HG (Figure 3B). In ObCRC subjects, the down-regulated XIST interacted with 264 DET while the up-regulated SNHG16 and AC109460.3 interacted with more than 10 DET. Other 3 DEL (LINC01184, STAG3L5P-PVRIG2P-PILRB, AP000317.2) had more or equal than 5 directed edges (Figure 3C).

**Figure 4:**
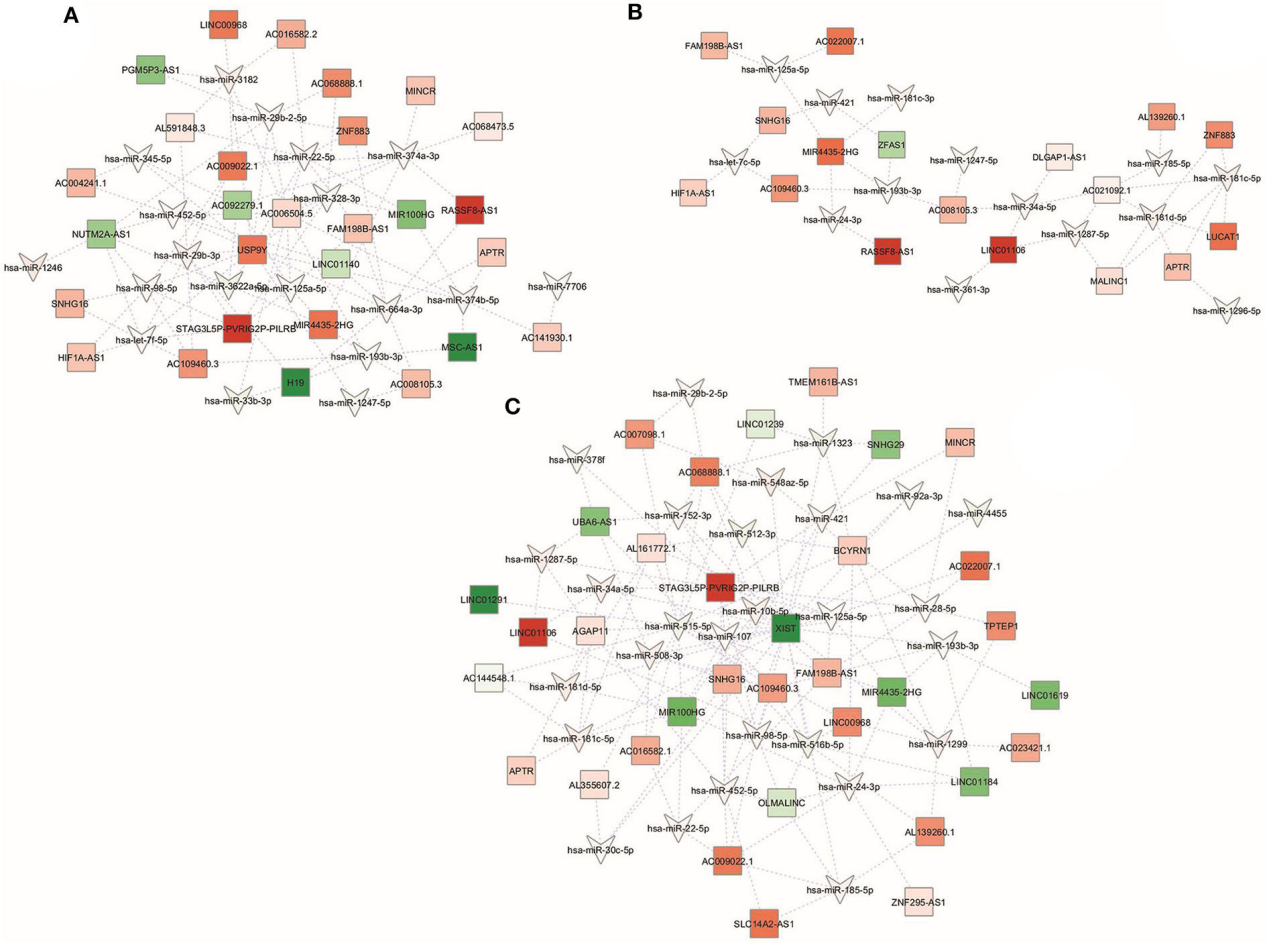
lncRNA-miRNA interaction networks. Interaction networks between deregulated lncRNAs (DEL; squares) and miRNA (DEM; V-shapes) in **(A)** NwCRC, **(B)** Ob, and **(C)** ObCRC patients in comparison to healthy lean subjects. Every node represents one gene, and each edge represents the interaction between genes. Shades of green and red indicate, respectively, down- or up-regulated DEL/DEM.

Since lncRNAs can bind to miRNAs to “communicate” with other RNA targets as well as to be reciprocally regulated by miRNAs (21), we then explored the ENCORI database for experimentally validated DEL-DEM interactions. As shown in Figure 4, DEL-DEM interaction networks in CRC patients, both lean and obese, displayed more interconnections than in obese individuals not affected by CRC. In particular, 95 relationship pairs between 28 DEL and 19 DEM were found in NwCRC patients, with the DEL USP9Y and AC006504.5 interacting with 12 and 11 DEM respectively; besides, hsa-miR-664a-3p and hsa-miR-22-5p were the top interaction DEM with 8 direct connections to DEL (Figure 4A). Likewise, in ObCRC subjects, 146 relationship pairs between 34 DEL and 28 DEM were found, with XIST and STAG3L5P-PVRIG2P-PILRB interacting with 23 and 11 DEM, respectively, and the top DEM hsa-miR-515-5p and hsa-miR-516b-5p interacted with 15 and 8 DEL, respectively (Figure 4C). Conversely, in Ob subjects only 37 relationship pairs between 16 DEL and 15 DEM were found, with AC021092.1 interacting with 5 DEM and hsa-miR-181d-5p and hsa-miR-181c-5p interacting with 5 DEL (Figure 4B).

### mRNA-miRNA-lncRNA Regulatory Networks

In order to identify novel key regulators in the transcriptional and post-transcriptional adipocyte reprogramming under obesity and CRC conditions, integrated lncRNA-miRNA-mRNA networks were constructed for each conditions taking into account and combining the interactions described between miRNA/mRNA, lncRNA/miRNA, and lncRNA/mRNA.

**Figure 5:**
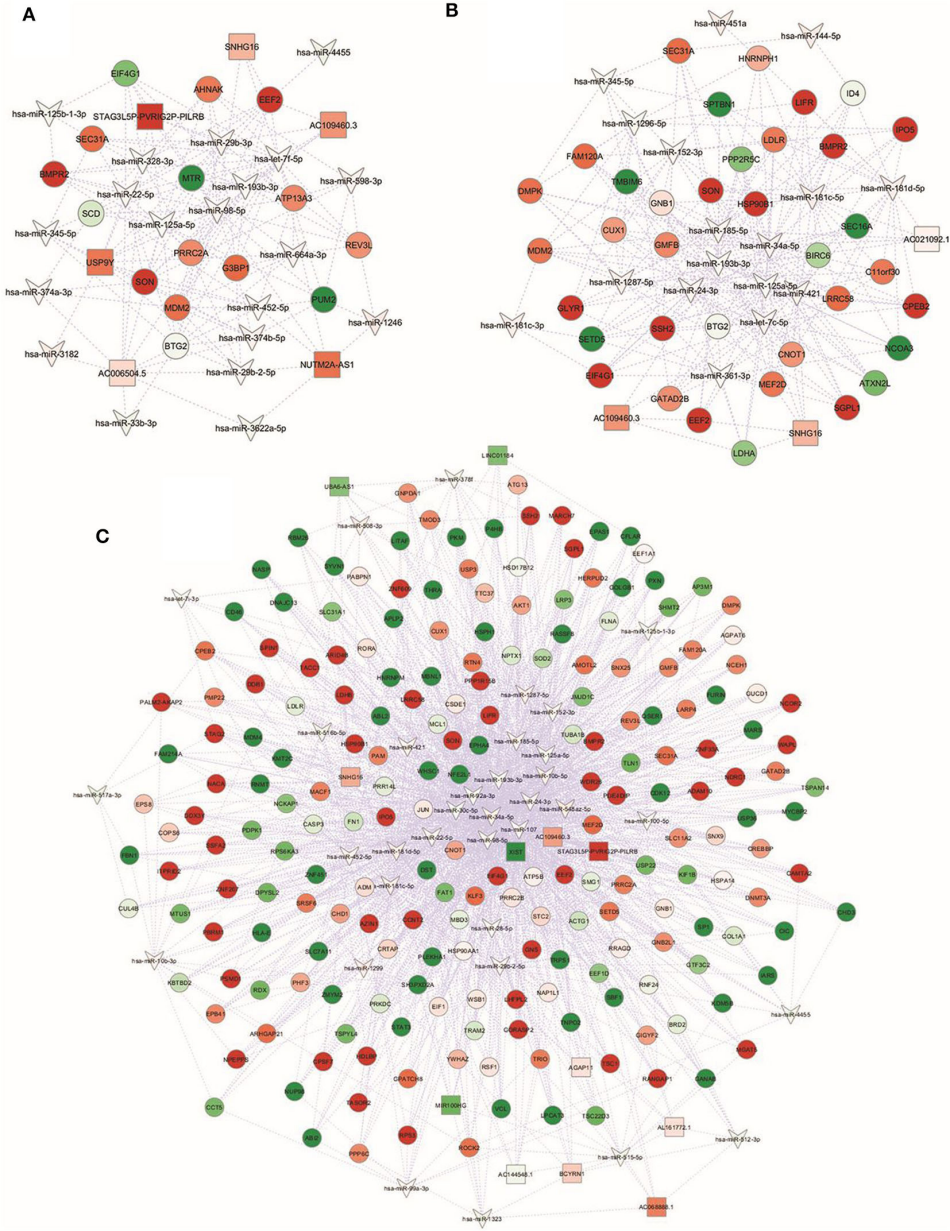
Global view of the miRNA-lncRNA-mRNA interaction networks. miRNAs are indicated with a V-shape, lncRNAs are indicated with squares, and mRNAs are indicated with circles. Only nodes with a number of directed edges ≥ 6 are shown. Shades of green and red indicate, respectively, down- or up-regulated DEM/DET/DEL. **(A)** NwCRC, **(B)** Ob, **(C)** ObCRC individuals in comparison to healthy lean subjects.

In this regard, it is reported that a stronger connectivity of RNA nodes in the network can reflect the importance of the biological functions of these RNAs in the network. Therefore, hub nodes with degree exceeding 5 represent key players in biological networks (43). Based on this criterion, different number and distribution of hubs, according to the RNA type, were identified in the three integrated networks. Specifically, we described 9 lncRNAs, 20 miRNAs, and 79 mRNAs hubs in the NwCRC network, 3 lncRNAs, 18 miRNAs, and 70 mRNAs hubs in the Ob network, and 10 lncRNAs, 36 miRNAs, and 308 mRNAs hubs in the ObCRC network, according to the higher complexity already described for the ObCRC condition in term of DEM-DET, DEL-DET, DEL-DEM interactions. Due to the complexity of the networks, only nodes with degree equal or higher than 6 are shown in Figure 5, whereas results description refers to the whole network. Focusing on ncRNAs, the most highly connected hubs in the NwCRC network were let-7f-5p, miR-98-5p, miR-193b-3p, miR-29b-3p, while SNHG16, and NUTM2A-AS1 had higher degrees compared with the other lncRNAs (Figure 5A). In the Ob network, miR-34a-5p, let-7c-5p, miR-24-3p, miR-193b-3p, and SNHG16, along with the novel lncRNA AC109460.3, were the most highly connected hubs ncRNAs (Figure 5B). Predominant nodes in the ObCRC network were miR-34a-5p, miR-107, miR-92a-3p, miR-24-3p, while the lncRNA XIST represents the main key interactor in the network (Figure 5C).

Searching for common key regulators, 23 genes were found to be the pivotal nodes in all networks, which include two miRNAs, miR-193b-3p, and miR-125a-5p, a known (SNHG16) and a novel (AC109460.3) lncRNA, and 19 mRNAs, indicating that these common elements and their interactors could be involved in relevant processes in obesity and CRC. Among the shared mRNA nodes, we found key players involved in the adipocyte transcriptional program (e.g., STAT3, RORA, CNOT1), in adipogenesis and lipogenesis processes (e.g., SEC31A, BMPR2) and in food intake and hypothalamic signaling (e.g., SON, PRRC2A, CUX1).

### Functional Enrichment Analysis of Networks-Related mRNA Targets

**Figure 6:**
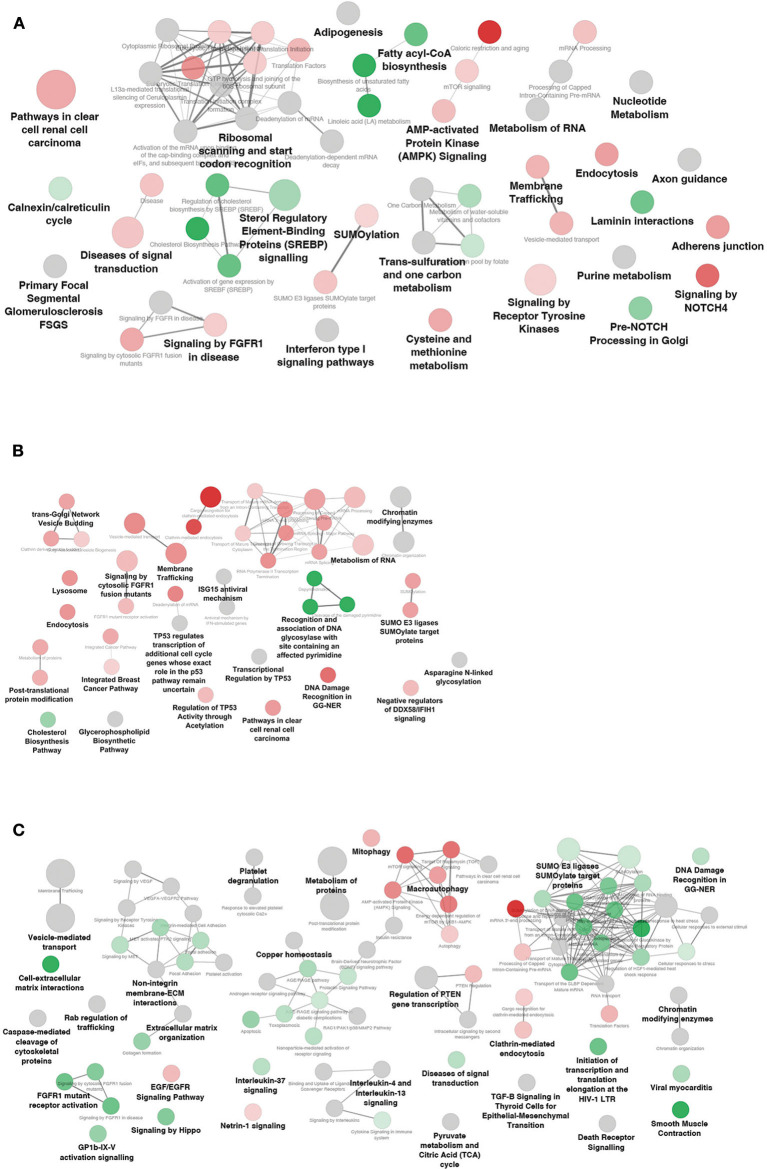
Functional enrichment analysis of differentially expressed mRNAs included in the networks. Significantly affected pathways in **(A)** NwCRC, **(B)** Ob, **(C)** ObCRC patients compared to lean healthy subjects. Each node represents a significantly enriched KEGG/Wiki/Reactome term, the diameter being proportional to the significance. Shades of green and red indicate that the node features > 50% down-regulated or up-regulated genes, respectively. Gray nodes indicate terms with equal contribution of up- down-regulated genes.

The biological function of a miRNA-lncRNA-mRNA network may be explained by the functions of the included target mRNAs. Thus, target genes of DEM and/or DEL found in the interaction networks of each subject group, were subjected to functional enrichment analysis combining different databases (KEGG, WikiPathways, and Reactome). The detailed list of terms, along with the genes involved in each term, are reported in Supplemental Table 3 and results are summarized in Figure 6.

In NwCRC patients numerous pathway terms associated with metabolic processes (e.g., *One-carbon metabolism, Purine metabolism, Cysteine/Methionine metabolism*), lipid metabolism (e.g., *Fatty acyl-coA biosynthesis, AMPK, and SREBP signaling*) and pathways involved in cancer (e.g., S*ignaling by FGFR1 in disease, Pathway in clear cell renal cell carcinoma*) were obtained (Figure 6A). While the cancer pathways mainly featured up-regulated genes, the lipid metabolism pathways mainly included down-regulated genes. As expected, also the obesity- associated network was enriched in terms related to lipid metabolism (e.g., *Cholesterol biosynthesis, Glycerophospholipid Biosynthetic Pathway*). Further, in Ob individuals, we found enriched cancer pathways shared with CRC lean subjects (e.g., *Signaling by FGFR1, Pathway in clear cell renal cell carcinoma, Integrated Breast Cancer Pathway*), or unique of obese condition, such as a TP53-related pathway, all induced (Figure 6B). Finally, the ObCRC network (Figure 6C) was primarily enriched by fundamental biological functions that are implicated in inflammatory signaling pathway (e.g., *Platelet degranulation, TGF-beta signaling, IL-4, and IL13 signaling*), tumor suppression and insulin sensitivity (e.g., *Regulation of PTEN gene transcription, Interleukin-37 signaling, Insulin resistance*), along with categories related to metabolism (e.g., *Pyruvate metabolism and Citric Acid cycle, AMPK signaling*) and cancer (e.g., *FGFR1 mutant receptor activation; signaling by VEGF*). Interestingly, in contrast to what observed for Ob and NwCRC networks, the majority of enriched categories featured under-expressed genes in ObCRC patients, with the exception, among others, of pathways related to energy metabolism (e.g., *mTOR signaling* and *AMPK signaling)*, to the growth factor EGF (e.g., *EGF/EGFR signaling pathway)* and to neuronal development (e.g., *Netrin-1 signaling*).

Finally, pathways related to type I interferon signaling (e.g., *Interferon type I signaling pathway, ISG15 antiviral mechanism, antiviral mechanisms by IFN- stimulated genes*) are shared by obese and CRC networks. Furthermore, all networks described showed dysregulated genes belonging to processes involved in RNA regulation (e.g., *metabolism of RNA*), endocytosis and vesicle-mediated transport (e.g., *Membrane trafficking, Vesicle budding, Endocytosis, Extracellular matrix organization*) and sumoylation (e.g., *SUMO E3 ligases SUMOylate target proteins*). Interestingly, in ObCRC patients we observed a predominant pathway repression state, again indicating that the interplay between obesity- and CRC results in a specific modulation of adipocyte transcriptional and post-transcriptional program.

### Validation by Using Real Time qPCR

**Figure 7:**
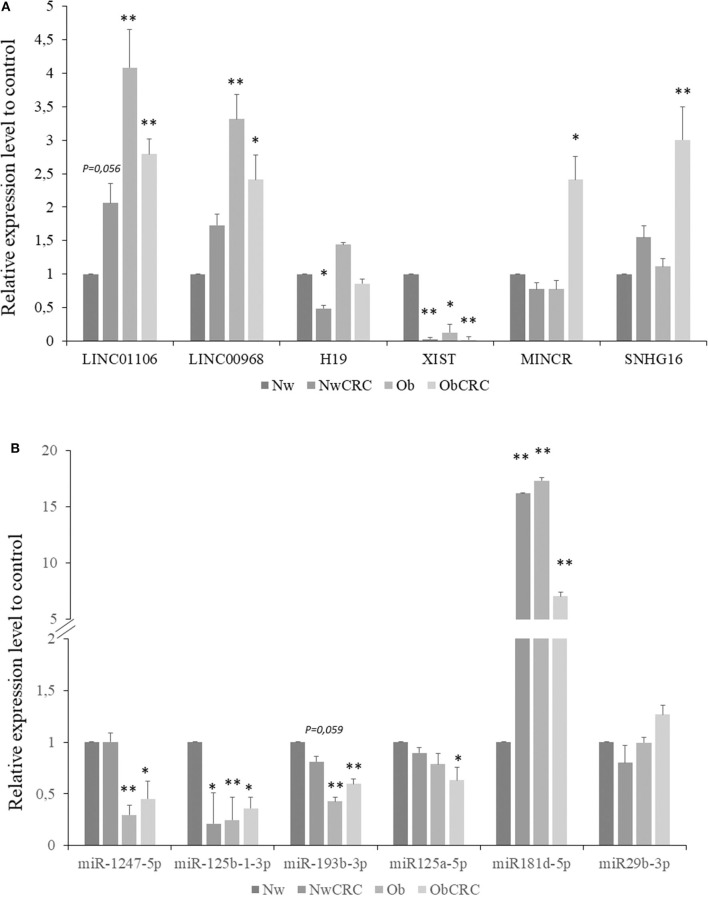
Validation by real-time qPCR of selected lncRNAs and miRNAs. Expression level of selected lncRNAs **(A)** or miRNAs **(B)** for NwCRC, Ob, and ObCRC subjects were normalized to healthy lean control. Statistical significance is indicated with * for *p* ≤ 0.05, and ** for *p* ≤ 0.005 *vs*. Nw control.

The expression levels of pivotal transcripts were validated by RT-qPCR. Candidate transcripts were selected among those DEL and DEM found to be shared between cancer and obese conditions (e.g., LINC01106, LINC00968, SNHG16, miR-125a-5p, miR-193b-3p, miR-1247-5p), along with those of ncRNAs specific for CRC or obese subjects (e.g., XIST, H19, MINCR, miR-29b, miR-125b-1-3p, miR-181d-5p), on the basis of their relevance in the described regulatory networks. As shown in Figure 7, the lncRNAs belonging to all categories of subjects (obese and CRC affected) were found to be significantly modulated compared to healthy lean subjects. Specifically, LINC01106 was significantly up-modulated in Ob and ObCRC, while H19 was specifically down-modulated in NwCRC patients. We failed to observe a significant up-regulation of LINC00698, MINCR and SNHG16 in NwCRC patients, although we confirmed their up-regulation in the other subject groups (Ob and ObCRC for LINC00698 and ObCRC for MINCR and SNHG16), according to RNASeq analysis (Figure 7A). Overall, RNASeq and qPCR data displayed a significant positive correlation (Rho = 0.829; *p* < 0.0001). Similarly, in the case of miRNAs (Figure 7B), qPCR analysis confirmed the down-modulation of miR-125b-1-3p in all conditions and miR-193b only in Ob and ObCRC, whereas the under-expression of miR-1247 and miR miR-125a-5p was validated in Ob and ObCRC groups or ObCRC group, respectively. We also reported an up-regulation of miR-181d-5p in both Ob and CRC affected subjects, although RNASeq data showed its over-expression in Ob subjects only. In contrast to what observed from RNASeq analysis, no differential expression of miR-29b-3p was found in all groups of subjects. Overall, although we did not achieve a complete correspondence between miRNA expression data from the two different techniques, qPCR and RNASeq results were significantly correlated (Rho = 0.6079; *p* = 0.0074).

## Discussion

The prevalence of obesity and obesity-associated diseases, including CRC, is in constant increase, accounting for a large portion of public health challenges. These multifactorial and complex disorders are strongly interconnected, although the mechanisms underlying the higher susceptibility to cancer development and the poorer cancer prognosis in obese individuals are still a matter of debate. Different components of the AT microenvironment, such as chronic inflammation, vascularity and fibrosis, altered levels of sex hormones, insulin resistance, are nowadays recognized as important determinants of CRC risk. Moreover, adipocytes release lipids acting as an energy reservoir for cancer cells, while the rapid expansion of AT in obesity produces hypoxia and promotes angiogenesis, favoring the tumor spread (7, 44, 45). Recent findings in epigenetics emphasized an important functional role of miRNAs, as well as of lncRNAs, in pathophysiological processes. The dysregulation of these transcripts, in fact, has been found in pathological conditions such as cancer and dysmetabolic disorders including obesity. In AT, miRNAs regulate all aspects of the adipocyte biology, including inflammation and adipokines production, metabolic responses, lipolysis and lipogenesis, adipogenesis and browning (9, 46). Likewise, the total number of lncRNAs identified in AT and found to modulate adipose function, is rapidly increasing (26, 47–49). Several studies reported the involvement of lncRNAs in adipogenesis and lipid metabolism (27, 50) as well as in AT function and development in mouse models (51, 52). Nevertheless, their implication in human adipocytes remains largely unknown. Likewise, no definitive conclusions regarding the molecular factors and the mechanistic processes underlying the relationships among obesity, AT dysfunction and CRC have been reached so far. To the best of our knowledge, this is the first comparative study that performed an integrated multi-omic analysis on human visceral adipocytes to assess how obesity, alone or combined to CRC, affects miRNA, and lncRNA expression and networks, as a potential mechanism linking obesity and CRC.

The expression of miRNAs of obese subjects with respect to lean individuals has been previously investigated in both VAT and subcutaneous AT (SAT), the two main fat depots that exhibit significant differences in anatomical, cellular and molecular features (6, 53). Heterogeneity of subjects (fat depots, BMI), type of samples (isolated adipocytes compared to adipose tissue), together with the use of different high-throughput techniques (arrays, RNA sequencing) has rendered difficult to identify a specific “miRNA signature” altered in obesity (9). In this regard, differences in miRNAs expression were observed when comparing visceral and subcutaneous fat (17, 54, 55), or isolated adipocytes and whole AT (56). In our study, we performed a whole analysis of miRNAs in human adipocytes isolated from the visceral fat. Among the miRNAs dysregulated in obese subjects compared to the normal weight controls, we found those involved in adipogenesis (e.g., let-7 family, miR-193b,−483-5p), in lipid metabolism (e.g., miR-181d), or in glucose and insulin metabolism (e.g., miR-34a-5p,−24-3p,−144-5p,−361-3p), previously described in different AT depots of obese subjects (9, 57–59), further supporting a role of these miRNAs in the functional alterations of adipocytes occurring in obesity. Additionally, in obese adipocytes we also reported the dysregulation of those miRNAs previously found to be involved in the regulation of immune response, adipokine secretion and inflammation (e.g., miR-125a-5p; −181 family, −193b) (15, 60, 61) or implicated in many aspects of carcinogenesis in several cancer types, including CRC (e.g., miR-34a, let7e-3p, −144-5p, −193b, −361-3p, −451a) (54, 62). Specifically, we found that miR-125a-5p and miR-193b-3p were downregulated in both obesity and CRC, in keeping with their previously reported down-regulation in VAT of obese subjects (63, 64), although contrasting results on miR-193b expression have been showed in human SAT (56, 64). Notably, we have previously described an up-regulation of the target genes of miR-193b (i.e., CCL2) and miR-125a-5p (i.e., STAT3), as an important mechanism underlying obesity-associated inflammation (29, 65), according to the literature (54, 56). Furthermore, we also report the characterization of 35 novel and 55 known lncRNAs in visceral adipocytes. An important property of lncRNAs is their cell- and tissue- specific expression (66). Therefore, the current annotation of lncRNAs is far from being complete. Alterations in the expression of some lncRNAs have been reported in both SAT and VAT, as important regulators of AT functions (26, 27, 67). In our study, we report the first analysis of lncRNAs in purified visceral adipocytes and this could explain the discrepancies observed with previous studies mainly conducted in whole AT (26, 27, 67). In general, we identified known and novel lncRNAs not previously described in other reports. Specifically, in obese subjects we found several lncRNAs (e.g., ZFAS1, LUCAT1, HIF1A-AS1, HOXB-AS3) already identified in the setting of different type of cancers, but not previously reported in human AT. Moreover, the lncRNA MIR3142HG, recently described as important mediator of the inflammatory response in Idiopathic Pulmonary Lung Fibroblasts positively regulating CXCL8 and CCL2 release (68), is specifically up-modulated in obesity. Notably, we previously reported an upregulation of both CCL2 and CXCL8 in adipocytes from Ob individuals (29), suggesting a role of this lncRNA in the AT inflammation. Other two lncRNAs, SNHG16, and LINC01106, were found to be upregulated in obesity, and this modulation was shared between obese and cancer conditions. In this regard, an abnormal expression of SNHG16 has been observed in multiple cancers and usually correlates with worse pathological features (69), while the novel lncRNA LINC01106 has been recently reported to be related to the overall survival of CRC patients by acting as inflammatory mediator in inflammatory bowel disease (IBD)-related CRC. This lncRNA showed also an intimate interaction with miR-193a in epithelial tissue from IBD and CRC patients (70).

Despite the well-known link between AT related inflammation and CRC development, no previous studies considered the expression of ncRNAs in the AT of CRC patients. When overlapping the data from NwCRC, Ob, and ObCRC individuals, the down-regulation of miRNAs, such as miR-193b-3p, miR-125a-5p, and miR-1247-5p, was found to be shared between cancer and obese conditions. Interestingly, both miR-193b-3p, and miR-1247-5p act as tumor suppressors in CRC or other types of cancer (71, 72), suggesting that their repression in AT from Ob and CRC individuals could have a potential pro-tumorigenic role. Beside common features, some ncRNAs are unique of tumor conditions. For instance, lncRNA H19, among others, was repressed only in NwCRC patients, with respect to healthy control. Interestingly, H19 has been described to play a role in obesity-induced cancer and to promote epithelial-mesenchymal transition of CRC, with a reported poor prognosis for cancer patients exhibiting H19 induction (73, 74). However, we observed an opposite expression in AT compared to cancer cells, suggesting a different role of this lncRNA in visceral adipocytes, that could potentially involve H19 target genes STAT3 and SPARC (75, 76). Indeed, we and others previously reported a key role of STAT3 and SPARC in AT dysfunctions both in obese (28, 65, 77) and CRC conditions (28, 65). Similarly, the lncRNA XIST is highly down-modulated in the AT from CRC group, although its up-regulation in CRC tissues and cell lines was reported (75, 78, 79). Remarkably, XIST can act as oncogene or tumor suppressor depending on the human malignancies (80) and was recently identified as a candidate in mediating glucose metabolism in glioma and contributing to cancer progression (81).

In this study, we not only identified some specific lncRNAs and miRNAs across the adipocyte genome, but we also described miRNA-lncRNA-mRNA interaction networks and the functional analysis of the pathways in which the target genes are involved. The target genes we identified in the networks were mainly enriched in several pathways, associated with metabolic processes, lipid and energy metabolism, inflammation, and cancer. Specifically, the SREBP pathway was remarkably inhibited in the NwCRC network, with implications not only on lipid metabolism but also on inflammation-mediated metabolic diseases, as well as on immune responses (82). Of note, the lncRNA SNHG16, that we have identified as a main hub of this network, has been reported to modulate the lipogenesis via regulation of SREBP2 expression (83), and to affect others genes involved in lipid metabolism (84). Another intriguing connection identified in Ob network is the upregulated TP53 transcriptional regulation pathway. The activation of this pathway has been previously observed in obesity and correlated to the release of inflammatory cytokines fueling cancer initiation and progression (85), thus potentially setting the basis for a more tumor-prone AT microenvironment in obese subjects. Furthermore, p53 in human AT was shown to be involved in insulin resistance, adipogenesis, lipid metabolism and nutrient sensing (86).

We also previously reported the influence of obesity on the adipocyte transcriptional program in CRC, with ObCRC subjects showing a higher number of dysregulated genes and processes (28). Likewise, in this study we observe a higher complexity of ObCRC network in terms of lncRNA and miRNA profiles. Interestingly, we describe in ObCRC patients the deregulation of fundamental biological functions that are mainly implicated in inflammatory signaling pathways, such as IL-37 and IL-13 signaling. In this regard, an increase expression of the cytokine IL-13, contributing to AT inflammation, has been reported to play an important role in obesity-related colon carcinogenesis (87), while IL-37 signaling has been described to play an inhibitory role in innate immune responses. In fact, it acts by reducing systemic and local inflammation, whereas its expression in SAT was negatively correlated with BMI (88). Other enriched categories in ObCRC network are: (i) TGF-beta signaling that has been reported to regulate multiple aspects of AT biology (i.e., vascularization, inflammation and fibrosis) (89), (ii) Netrin-1 signaling, recently described to play a role in tissue regulation outside the nervous system, specifically in tumor development (i.e., angiogenesis and inflammation) and (iii) PTEN regulation, for which a dual role as tumor suppressor and metabolic regulator has been reported (90). Finally, the networks described in all subject groups were enriched in: (i) type I IFN signaling, recently identified as essential in the regulation of metabolism and in maintaining AT function (91), (ii) SUMOylation, a post-translational modification mechanism that plays an emerging role in cellular metabolism and metabolic disease (92) and (iii) pathways involved in RNA metabolism, as expected. The identification of these pathways in both obese and cancer groups strongly points to the local metabolic alterations in AT as key element in colorectal carcinogenesis.

Additionally, pathways related to membrane trafficking, vesicle budding and endocytosis processes were also found to be dysregulated in both obesity and CRC networks. In this regard, it is worth to note that in addition to act locally, adipocytes influence and communicate with distant organs and tissues, by releasing bioactive molecules, such as triglycerides, adipokines, cytokines, and free fatty acids (93). This ability allows even tumors with no direct contact with AT to be affected by obesity, as indicated by epidemiological studies linking obesity with several types of cancers (94). Among adipocytes products that could sustain cancer cell growth, circulating miRNAs, both naked or associated to exosomes, may regulate the function of the immune system and distant organs and could potentially be used as biomarkers of diagnosis and prognosis of obesity and cancer (15). Likewise, exosomal lncRNAs have been shown to promote angiogenesis, cell proliferation and drug resistance and can be found in several body fluids, being highly stable, thus considered potential tumor biomarkers (95).

In conclusion, the importance of understanding the role of lncRNAs and miRNAs in AT of obese and CRC affected subjects extends beyond the description of gene regulation mechanisms. The results obtained in this study, through a multi-omics approach and computational analysis, contribute to the identification of candidate genes, ncRNAs and their regulatory networks relevant to many AT biological processes, although the direct causality remains to be established, requiring further experimental and functional studies. Nonetheless, the identification of AT miRNAs and lncRNAs as key components of interrelated processes and pathways may not only better define their role in human AT, but also identify promising mechanism-based targets, to disrupt the relationship between obesity, metabolic dysregulation, and cancer, potentially improving intervention and treatment plans.

## Data Availability Statement

The datasets generated for this study can be found in the NCBI Sequence Read Archive (SRA) (http://www.ncbi.nlm.nih.gov/sra) (PRJNA632999, PRJNA508473).

## Ethics Statement

The studies involving human participants were reviewed and approved by the institutional review board of Istituto Superiore di Sanità. The patients/participants provided their written informed consent to participate in this study.

## Author Contributions

RV and BS isolated adipocytes from human visceral adipose tissue biopsies. AB prepared samples for RNA Sequencing and performed real-time qPCR for gene validation. ST, AM, EC, and PM performed bioinformatics and statistical analyses of RNASeq data. ST and AM provided intellectual input throughout the study. MD and SG provided substantial contributions to the conception of the work as well as interpretation of data and manuscript writing. All authors contributed to the article and approved the submitted version.

## Conflict of Interest

The authors declare that the research was conducted in the absence of any commercial or financial relationships that could be construed as a potential conflict of interest.
