# Supplementary material for: Integrated Transcriptome Analysis of Human Visceral Adipocytes Unravels Dysregulated microRNA-Long Non-coding RNA-mRNA Networks in Obesity and Colorectal Cancer
Source: Front Oncol. 2020 Jul 2;10:1089. doi: 10.3389/fonc.2020.01089 (PMC7351520; doi:10.3389/fonc.2020.01089)
Supplement: Supplementary file 1 [file Table_1.pdf]

Supplemental Table 1. List of lncRNA primers selected for real time qPCR analysis

	**Forward (5'->3')**	**Reverse (5'->3')**
**LINC01106**	TCCTTGAGCTGAACGTCTTG	CCCATAAGCAGGCAGAAGAA
**LINC00968**	CTGGCACCTCATGTCTCTTTAG	TCAGAGCCTCTCTGTAGCTTAG
**SNHG16**	GACCAAGGAGGGACTGTTTAAG	CCTGGACAGCAGGAATTAAGAG
**XIST**	CAGTCAGGAGAAAGAAGTGGAG	GACAGAGGTGGAAAGGCTAAA
**H19**	AGCGGGTCTGTTTCTTTACTT	AGCTGGGTAGCACCATTTC
**MINCR**	GGTCTCAAGTCTACTGGCTTTC	ATGCACTCACAGGGTTATGG
**GUSB**	AAGACGCACTTCCAACTTGA	GATGACATCACCGTCACCA
